# Sexual risk behaviour among people living with HIV according to the biomedical risk of transmission: results from the ANRS-VESPA2 survey

**DOI:** 10.7448/IAS.19.1.20095

**Published:** 2016-01-08

**Authors:** Marie Suzan-Monti, Nicolas Lorente, Baptiste Demoulin, Fabienne Marcellin, Marie Préau, Rosemary Dray-Spira, France Lert, Bruno Spire, the ANRS-VESPA2 Study Group

**Affiliations:** 1 INSERM, UMR 912 (SESSTIM), Marseille, France; 2 Aix Marseille Université, UMR_912, IRD, Marseille, France; 3 Observatoire Régional de la Santé Provence-Alpes-Côte d'Azur, Marseille, France; 4 Groupe de Recherche en Psychologie Sociale, Université Lyon 2, Lyon, France; 5 INSERM, UMR_S1136, Pierre Louis Institute of Epidemiology and Public Health, Research Team in Social Epidemiology, Paris, France; 6 Sorbonne Universités, UPMC Univ Paris 06, UMR_S1136, Pierre Louis Institute of Epidemiology and Public Health, Research Team in Social Epidemiology, Paris, France; 7 Centre de recherche en épidémiologie et santé des populations, INSERM U1018, Villejuif, France

**Keywords:** HIV, successful ART, biomedical risk of HIV transmission, sexual risk behaviour

## Abstract

**Introduction:**

People living with HIV (PLHIV) on antiretroviral therapy (ART), with sustained undetectable viral load (sUVL) and no history of sexually transmitted infections for at least six months, are considered to have a low risk of HIV transmission (LRT). We aimed to characterize, in a representative sample of French PLHIV, the sexual behaviour of LRT PLHIV compared with non-LRT PLHIV.

**Methods:**

The cross-sectional ANRS-VESPA2 survey was conducted on adult PLHIV attending French hospitals in 2011. The LRT PLHIV group included participants with sUVL and no sexually transmitted infection for at least 12 months. Socio-behavioural and medical data were collected. Chi-square tests helped compare sexual risk indicators between LRT and non-LRT PLHIV. The survey's retrospective nature allowed us to perform complementary category-based analyses of LRT PLHIV according to whether they had sUVL for at least 18, 24 or 36 months in three socio-epidemiological groups: men who have sex with men (MSM), other men and women.

**Results:**

Analysis included 2638 PLHIV diagnosed >12 months with available viral load data. The proportion of LRT PLHIV varied from 58% (≥12 months sUVL) to 38% (≥36 months sUVL). Irrespective of sUVL duration, we found the following: 1) LRT men (MSM and other men) were more likely to report having no sexual partner than their non-LRT counterparts. Among men having sexual partners in the previous 12 months, no significant difference was seen between LRT and non-LRT men in the number of sexual partners. LRT women were less likely to report having more than one sexual partner than non-LRT women; 2) LRT MSM were more likely to report being in sexually inactive couples than their non-LRT counterparts; 3) among sexually active participants, no difference was observed between LRT and non-LRT PLHIV concerning condom use with their serodiscordant steady partner or with their most recent casual sexual partners.

**Conclusions:**

LRT PLHIV with sUVL ≥12 months did not report more sexual risk behaviours than their non-LRT counterparts. Because the same result was obtained for those having a sUVL ≥36 months, the hypothesis of increased sexual risk behaviour over time in PLHIV meeting non-transmission biomedical criteria is not supported.

## Introduction

Antiretroviral therapies (ART) are now part of combination HIV prevention strategies, as their efficacy in decreasing blood plasma viral load (VL) has led to a dramatic reduction in HIV-1 sexual transmission among heterosexual serodiscordant couples [1–4]. In 2008, the Swiss Federal Commission for HIV/AIDS stated that HIV-positive heterosexual individuals on effective ART – individuals having an undetectable plasma VL for at least six months with no sexually transmitted infection (STI) during the same period – can be considered sexually non-infectious [5]. This statement has led to considerable debate about possible sexual risk disinhibition or risk compensation in this population [6–10], offsetting the benefits of current biomedical HIV prevention strategies. This issue is particularly important since the number of new HIV diagnoses continues to grow in the most vulnerable populations, especially men who have sex with men (MSM). This is the case in France where a 14% increase was observed between 2011 and 2013 in this population [11].

Systematic reviews or meta-analyses of the association between ART, risk perception and sexual behaviour show no or short-term increases in sexual risk behaviour among people receiving ART [12–15]. Meta-analysis by Crepaz *et al*. demonstrated no higher rates of condomless sex between people living with HIV (PLHIV) on ART with a detectable VL and with undetectable VL (UVL). However there was a high prevalence of condomless sex in persons – with known or unknown HIV status – who believed that being on ART or having an UVL protects against HIV transmission or who were less concerned about engaging in unsafe sex because of ART availability. A review of recent findings also supported the relationship between treatment-related optimistic beliefs and HIV transmission risk [16]. Recent results from the Swiss HIV Cohort Study showed increased condomless sex with stable partners, after 2008, among MSM and heterosexual ART-treated PLHIV with a UVL. It was suggested that this increase was possibly the consequence of PLHIV believing that HIV treatment was a sufficient prevention strategy [17]. More recently, a cross-sectional study among a nationally representative sample of US PLHIV engaged in care showed that the majority did not engage in sexual risk behaviour and that half of those who did had a detectable VL during the previous 12 months [18]. The heterogeneity of results published in the literature reflects the diversity of the study designs they come from (longitudinal studies, cohorts, cross-sectional surveys), the diversity of the studied populations (heterosexual couples, MSM, drug users) and potential cross-cultural differences. However results from behavioural cohorts or from representative samples of PLHIV are scarce. We used data collected during the ANRS-VESPA2 survey to analyze, for the first time, the evolution over time of sexual risk behaviour in three distinct socio-epidemiological groups: MSM, women and other men, according to the biomedical criterion of HIV transmission risk.

The cross-sectional ANRS-VESPA2 survey was performed among a nationally representative sample of adult PLHIV living in France in 2011 to provide information on various aspects of their conditions, including socio-demographic, epidemiological and health status data as well as HIV medical care characteristics. More than 93% of patients were receiving ART, and among them approximately 57% had a CD4 cell count >500 cells/mm^3^, whereas 88.5% had a controlled VL. The present study aimed to analyze, in this representative sample, the sexual risk behaviour of ART-treated PLHIV meeting the biomedical criteria for low HIV transmission risk (LRT), defined in the present study as having an undetectable VL for at least 12 months and no STI in the previous 12 months, compared with their non-LRT counterparts. The goal was to determine whether less infectious individuals engaged more in sexual risk behaviour. Despite its cross-sectional nature, the ANRS-VESPA2 design allowed us to retrospectively collect participants’ medical data from the French electronic database Nadis^®^, in turn enabling us to carry out complementary analyses by categories of LRT PLHIV according to different durations of UVL. Our working hypothesis was that successful and sustained VL control does not translate into increased sexual risk behaviour in PLHIV.

## Methods

### Design and setting

The national cross-sectional ANRS-VESPA2 survey took place from April 2011 to January 2012 in 68 HIV care services in French hospitals. A representative sample of 3022 patients was included in the survey after patients provided written informed consent. Patients were drawn randomly from among 9098 eligible patients (i.e. >18 years old, HIV-diagnosed longer than six months, living in France for more than six months and attending participating outpatient services at the time of the study). Independent trained interviewers administered a face-to-face questionnaire to collect data about patients’ socio-demographic characteristics, different aspects of their lives with HIV, their social trajectory during the course of the disease and their sexual behaviours. Medical staff completed a questionnaire about patients’ health status, HIV history, co-morbidities and all prescribed treatments. Data were weighted and calibrated to be representative of the entire population of PLHIV followed-up on in French hospitals in 2011. To this end, individual weights were computed accounting for both the unequal probability of random selection and the heterogeneous rates of non-participation between PLHIV subgroups. A comprehensive description of the survey methodology can be found elsewhere [19]. The ANRS-VESPA2 study was approved by the Commission Nationale de l'Informatique et des Libertés, the French data protection authority (approval number DR-2010-368).

### Participants

The analysis included 2638 PLHIV of the 3022 patients enrolled in the VESPA2 survey, HIV-diagnosed >12 months and with available data on VL status (i.e. detectable VL at the time of the survey, or with known duration of sustained UVL (sUVL) for those who had achieved it). We retrospectively defined the duration of sUVL for each participant using medical data. Three socio-epidemiological groups were created based on participants’ response to the question “Would you define yourself as heterosexual/bisexual/homosexual/transgender?” as follows: 1) MSM (self-identified as homosexual, bisexual or heterosexual men reporting at least one male sexual partner); 2) other men (self-identified as heterosexual reporting only female partners); and 3) women (irrespective of sexual identity or behaviour).

No significant differences were observed between excluded (*n*=384) and included study sample participants (*n*=2638) regarding socio-epidemiological group, CD4 cell count and VL at most recent assessment and disease clinical stage. Participants were defined as LRT if they were receiving ART, had a sUVL ≥12 months and reported no STI in the previous 12 months (*n*=1419). This 12 month criterion was chosen to define LRT, instead of the six months used in the Swiss statement, based on the STI item in our study questionnaire.

### Variables

#### Duration of sustained undetectable VL

The retrospective nature of the ANRS-VESPA2 survey helped us define different sUVL groups according to sUVL duration: ≥12, ≥18, ≥24 or ≥36 months, which in turn enabled us to carry out complementary analyses to observe whether outcomes of participants in a more restrictive definition of sUVL (e.g. sUVL ≥36 months) were different from those in a less restrictive definition of sUVL (e.g. sUVL ≥12 months).

#### Sexual risk behaviour outcome

Sexual risk behaviour was evaluated using the following three variables as risk proxies: 1) total number of sexual partners in the previous 12 months (categorized into *no partner*, *1 to 20* and *>20 partners* for MSM; *no partner*, *1* or *>1* for other men and women); 2) condomless vaginal/anal intercourse with a serodiscordant steady partner in the previous 12 months; and 3) condomless vaginal/anal sex during the most recent encounter with a non-HIV-positive (i.e. HIV-negative or unknown status) casual partner. Participants reporting having a steady partner for ≥12 months were considered to be in a stable couple. Categorizing the number of sexual partners in the previous 12 months was used to obtain a homogeneous distribution of the sample size in each socio-epidemiological group considered. The range of partner numbers differed greatly between MSM, other men and women (median [CI] 6 [2 to 20], 2 [1 to 4] and 2 [1 to 3], respectively).

#### Condomless sex

For participants having a serodiscordant steady partner, condomless sex (“yes”) was defined as inconsistently or never using condoms (versus “no,” i.e., always using condoms) during vaginal/anal intercourse in the previous 12 months. When considering the most recent encounter with a non-HIV-positive (i.e. HIV-negative or unknown status) casual partner, condomless sex was defined as no condom use (“yes” versus “no”) during vaginal/anal intercourse.

### Statistical analysis

Three sexual risk behaviour proxies were considered: 1) the number of sexual partners for each participant; 2) condom use during anal or vaginal intercourse within serodiscordant couples in the previous 12 months; and 3) condom use during anal or vaginal intercourse with the most recent non-HIV-positive casual partner. Sexual risk behaviour was evaluated by comparing these risk proxies between LRT and non-LRT patients for the three socio-epidemiological groups: MSM, other men and women. Chi-square tests were used for all comparisons, with a significance threshold of 5%, and a 10% threshold to reflect marginal significance. The same analyses were performed using different durations of sUVL among the LRT PLHIV: ≥18, ≥24 and ≥36 months. In addition, a sensitivity analysis was performed after removing the criterion “absence of STIs in the previous 12 months” for PLHIV at “low risk of transmission,” since STIs were self-reported, with no corresponding medical data. Another analysis compared the LRT PLHIV in each socio-epidemiological subgroup with their non-LRT counterpart PLHIV on treatment. The latter were either virally suppressed and had experienced an STI in the previous 12 months or were not virally suppressed for the full time period considered. All analyses were performed on weighted and calibrated data. Stata/SE 12.1 for Windows (StataCorp LP, College Station, TX, USA) software was used for the analyses.

## Results

Among the 2638 PLHIV included in the study, 1163 were MSM (44.1%), 828 women (31.4%) and 647 other men (24.5%). Table 1 shows the main patient characteristics.

**Table 1:** Main characteristics of VESPA2 survey participants, diagnosed with HIV >12 months, with data on their viral load (*n*=2638)

	MSM^a^ *n*=1163 *n* (%) median (IQR)	Women *n*=828 *n* (%) median (IQR)	Other men^a^ *n*=647 *n* (%) median (IQR)	*p*
Age	49 (42 to 55)	44 (36 to 50)	50 (45 to 56)	<10^−3^
Education – secondary school or above	60.6	34.8	38.8	<10^−3^
Time since HIV diagnosis (years)	14.3 (6.6 to 20.3)	10.8 (6.3 to 18.9)	13.3 (7.3 to 20.6)	0.002
Time on ART (years)	10.3 (3.5 to 14)	7.6 (4.1 to 12.2)	9.7 (4.4 to 13.8)	<10^−3^
CD4 cell count at most recent assessment				
<200 cells/mm^3^	52 (3.5)	42 (4.9)	49 (6.1)	0.008
200 to 350	149 (12.6)	125 (13.2)	98 (19.4)	
350 to 500	243 (22.1)	124 (24.5)	195 (23.8)	
>500	715 (61.8)	353 (57.4)	484 (50.7)	

a MSM, men who have sex with men; other men, men who only have sex with women. IQR, interquartile ratio; ART, antiretroviral therapy.

Women were younger than MSM and other men with, respectively, a median (interquartile range, or IQR) age of 44 (36 to 50) years versus 49 (42 to 55) and 50 (45 to 56). They were also less likely to have an educational level higher than secondary school (34.8% versus 60.6% and 38.8%, respectively). Overall, median time since HIV diagnosis and on ART was >10 years and >7 years, respectively. Over 50% of the study sample had CD4 cell counts >500 cells/mm^3^ at their most recent assessment. Most patients were receiving ART at the time of the survey (*n*=2498, 93.6%).

**Figure 1:**
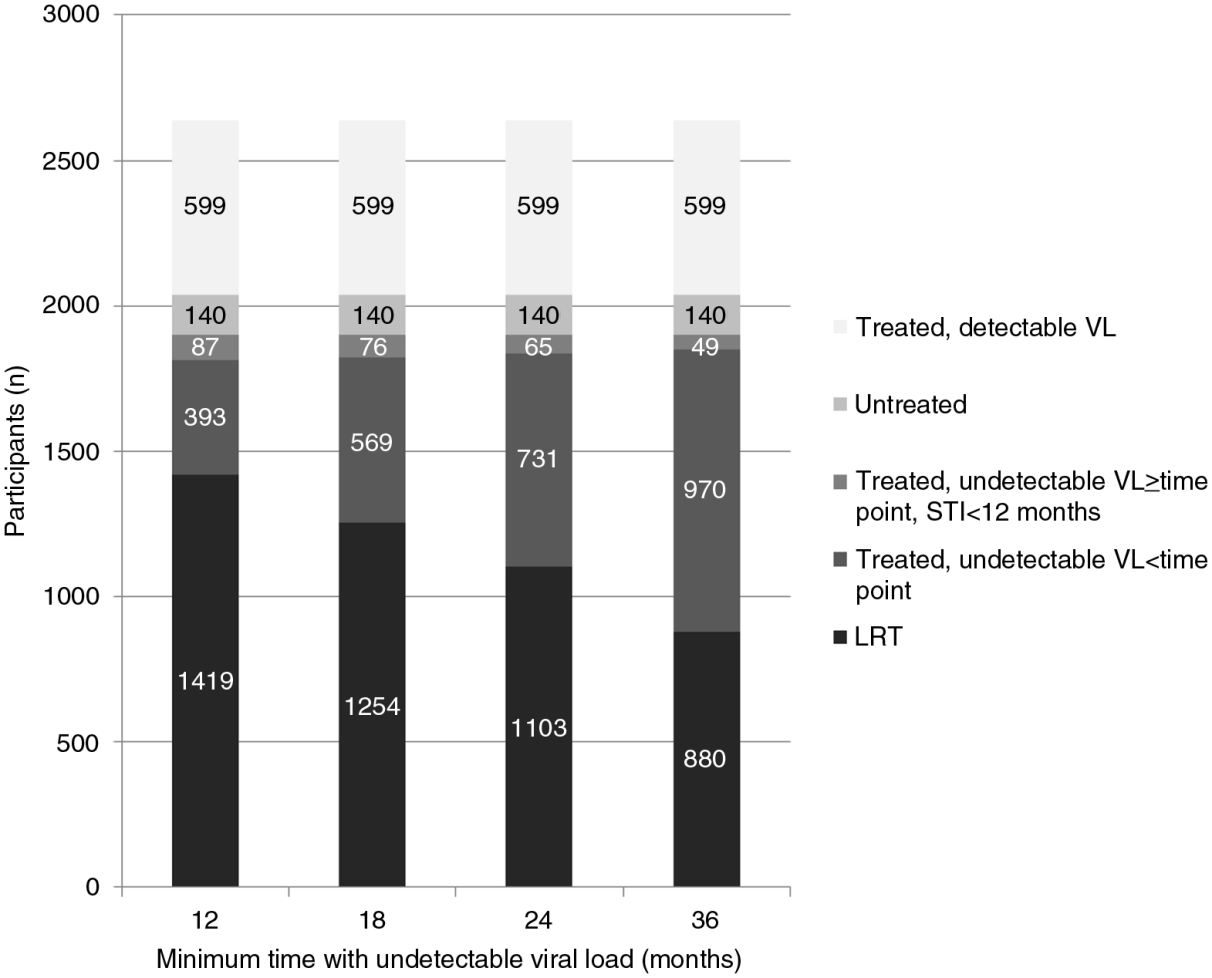
Distribution of participants among the study sample according to antiretroviral therapy treatment, virological status and according to minimum time (months) defining sustained undetectable viral load for participants with low HIV transmission risk (*n*=2638).

Overall, 58.1% (*n*=1419) of the study sample was considered to have a low risk of HIV transmission (LRT) for a sUVL of 12 months. Restricting the definition of sUVL among LRT PLHIV reduced this proportion as follows: 52.2% (*n*=1254) for sUVL ≥18 months, 46.8% (*n*=1103) for ≥24 months and 38.2% (*n*=880) for ≥36 months (Figure 1).

Patients considered not to have an LRT were either untreated (6.4%), were receiving ART and had a detectable VL (19.4%), were on ART and had sUVL but for a duration less than each of the four specific durations under consideration (12.5%, 18.8%, 24.7% and 33.8% for <12, <18, <24 and <36 months of sUVL, respectively) or were on ART with sUVL for a duration greater than each of the four specific durations under consideration but reported at least one STI in the previous year (3.6%, 3.2%, 2.7% and 2.2% for ≥12, ≥18, ≥24 and ≥36 months of sUVL, respectively).

**Figure 2:**
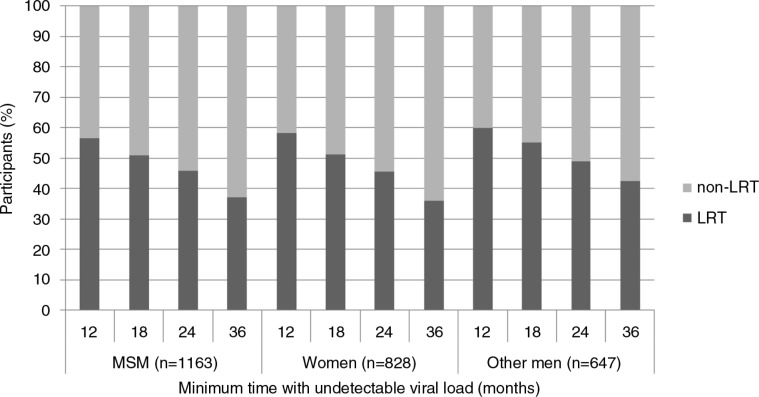
Distribution of participants among the study sample (*n*=2638) according to whether they had low HIV transmission risk (LRT) or not, within each socio-epidemiological group, and the minimum time (months) defining sustained undetectable viral load for LRT participants.

The proportion of LRT versus non-LRT participants in the three groups considered (i.e. MSM, women and other men) according to time is detailed in Figure 2.

Table 2 compares the total number of sexual partners in the previous 12 months between LRT and non-LRT PLHIV within each socio-epidemiological group according to the different definitions of sUVL. LRT MSM with sUVL for ≥12 or ≥18 months were significantly more likely to report no partners than their non-LRT counterparts (*p*=0.01 and *p*=0.007, respectively), but these differences were no longer significant for MSM with an LRT for the ≥24- or ≥36-month definitions of sUVL. Among MSM with sexual partners in the previous 12 months, no significant difference in the number of sexual partners could be observed between LRT and non-LRT participants. Irrespective of the definition of sUVL considered, LRT women were less likely to have more than one partner than their non-LRT counterparts. Other LRT men with sUVL defined as ≥36 months were significantly more likely to have one or more partners than their non-LRT counterparts (*p*=0.02). Among other men with sexual partners in the previous 12 months, no significant difference could be observed in the number of sexual partners between LRT and non-LRT participants.

**Table 2:** Total number of sexual partners in the previous 12 months according to the biomedical risk of HIV transmission (*n*=2638)

		Minimum time with undetectable viral load											
		
		12 months			18 months			24 months			36 months		
					
Group	Number of partners^a^	LRT (%)^b^	Non-LRT (%)^b^	p_a__vs.__(b+c)_^c^ p_b__vs.__c_^d^	LRT (%)^b^	Non-LRT (%)^b^	p_a__vs.__(b+c)_^c^ p_b__vs.__c_^d^	LRT (%)^b^	Non-LRT (%)^b^	p_a__vs.__(b+c)_^c^ p_b__vs.__c_^d^	LRT (%)^b^	Non-LRT (%)^b^	p_a__vs.__(b+c)_^c^ p_b__vs.__c_^d^
MSM (*n*=1163)	None^(a)^ 1 to 20^(b)^ >20^(c)^	24 62 14	17 63 20	0.01 ns^e^	25 61 14	18 63 19	0.007 ns	24 62 14	19 63 18	ns ns	22 63 15	20 62 18	ns ns
Women (*n*=828)	None^(a)^ 1^(b)^ >1^(c)^	38 57 5	38 51 11	ns 0.01	37 58 5	39 51 10	ns 0.02	38 57 5	38 52 10	ns 0.05	40 54 6	38 52 10	ns ns
Other men (*n*=647)	None^(a)^ 1^(b)^ >1^(c)^	26 56 18	30 52 18	ns ns	25 57 18	30 51 19	ns ns	24 56 20	31 52 17	ns ns	21 58 21	32 51 17	0.02 ns

a Number of partners: total number of sexual partners in the previous 12 months

b %, weighted percentage of patients

c individuals having no partner in the previous 12 months (a) were compared with those who had at least one sexual partner (b+c)

d computed on individuals reporting at least one sexual partner in the previous 12 months

e ns, not significant, *p*>0.10; LRT, low risk of HIV transmission.

In the study sample, 1038 (39.3%) patients reported being in stable serodiscordant couples. Table 3 compares condomless sex for LRT and non-LRT PLHIV with a serodiscordant steady partner, during vaginal/anal intercourse among sexually active couples, and sexual abstinence within couples, for the previous 12 months.

**Table 3:** Sexual practices in serodiscordant couples in the previous 12 months (*n*=1038)

		Minimum time since undetectable viral load											
		
		12 months			18 months			24 months			36 months		
					
Group	Variable	LRT (%)^a^	Non-LRT (%)^a^	p_(a+b)__vs.__c_^b^ p_a__*vs*__b_	LRT (%)^a^	Non-LRT (%)^a^	p_(a+b)__vs.__c_^b^ p_a__*vs*__b_	LRT (%)^a^	Non-LRT (%)^a^	p_(a+b)__*vs*__c_^b^ p_a__*vs*__b_	LRT (%)^a^	Non-LRT (%)^a^	p_(a+b)__vs.__c_^b^ p_a__*vs*__b_
MSM (*n*=382)	Sexually inactive couples^c^^(c)^	25	17	0.10	26	16	0.05	26	17	0.08	27	18	0.06
	Condomless sex^d^												
	No^(b)^	63	65		62	66		63	65		64	63	
	Yes^(a)^	12	18	ns^e^	12	18	ns	11	18	ns	9	19	0.07
Women (*n*=338)	Sexually inactive couples^(c)^	15	9	ns	15	10	ns	15	10	ns	15	11	ns
	Condomless sex												
	No^(b)^	52	60		51	61		51	60		55	55	
	Yes^(a)^	33	31	ns	34	29	ns	34	30	ns	30	34	ns
Other men (*n*=318)	Sexually inactive couples^(c)^	15	11	ns	14	12	ns	14	13	ns	12	14	ns
	Condomless sex												
	No^(b)^	68	69		69	67		69	67		70	66	
	Yes^(a)^	17	20	ns	17	21	ns	17	20	ns	18	20	ns
Total (*n*=1038)	Sexually inactive couples^(c)^	18	12	0.03	18	13	0.07	18	14	ns	18	14	ns
	Condomless sex												
	No^(b)^	61	65		60	64		60	64		63	62	
	Yes^(a)^	21	23	ns	22	23	ns	22	22	ns	19	24	ns

a %, weighted percentage of patients

b sexually active individuals (a+b) were compared to sexually inactive individuals (c)

c sexually inactive couples, no sexual intercourse with any type of partner

d condomless sex, unprotected anal or vaginal intercourse with a serodiscordant steady partner in the previous 12 months

e ns, not significant, *p*>0.10; LRT, low risk of HIV transmission.

In sexually active couples among each socio-epidemiological group, no significant differences were observed between LRT and non-LRT PLHIV regarding condom use with a serodiscordant steady partner, irrespective of the different definitions of sUVL for LRT participants. The exception was LRT MSM with sUVL for ≥36 months who showed a trend towards significantly less unprotected intercourse than their non-LRT counterparts (*p*=0.07). LRT MSM with sUVL for ≥18 months were more likely to engage in sexual abstinence than their non-LRT counterparts (*p*=0.05), and a trend towards significance was observed when considering LRT MSM with sUVL for ≥24 or ≥36 months (*p*=0.08 and 0.06, respectively). No significant difference was observed for this between LRT and non-LRT women and other men.

Less than one-third of PLHIV of the study sample (*n*=746) reported having a non-HIV-positive casual partner in the previous 12 months. Among these, the only trend observed was towards a lower proportion of other LRT men with sUVL for ≥24 months reporting condom use during their most recent sexual encounter with a non-HIV-positive casual partner when compared with their non-LRT counterparts (*p*=0.09, Table 4).

**Table 4:** Condomless sex with the most recent non-HIV-positive casual partner (*n*=746)

		Minimum time since undetectable viral load											
		
		12 months			18 months			24 months			36 months		
					
Group	Variable: Condomless sex^a^	LRT (%)^b^	Non-LRT (%)^b^	*p*	LRT (%)^b^	Non-LRT (%)^b^	*p*	LRT (%)^b^	Non-LRT (%)^b^	*p*	LRT (%)^b^	Non-LRT (%)^b^	*p*
MSM (*n*=526)	No	93	90		93	90		92	91		92	91	
	Yes	7	10	ns^c^	7	10	ns^c^	8	9	ns^c^	8	9	ns^c^
Women (*n*=89)	No	89	89		89	89		92	83		90	84	
	Yes	11	11	ns	11	16	ns	8	17	ns	10	16	ns
Other men (*n*=131)	No	84	94		82	94		82	94		85	91	
	Yes	16	6	ns	18	6	0.10	18	6	0.09	15	9	ns
Total (*n*=746)	No	91	90		90	90		90	90		90	90	
	Yes	9	10	ns	10	10	ns	10	10	ns	10	10	ns

a condomless sex, unprotected anal or vaginal intercourse

b %, weighted percentage of patients

c ns, not significant, *p*>0.10; LRT, low risk of HIV transmission; MSM, men who have sex with men.

The robustness of the results was confirmed after adjustment for age, educational level, income and time since diagnosis (data not shown). Moreover the results were confirmed by two sensitivity analyses. The first was performed after removing the criterion “absence of STIs in the previous 12 months” for PLHIV defined as being at low risk of transmission, since STIs were self-reported, with no corresponding medical data. The second compared the LRT PLHIV in each socio-epidemiological subgroup with their non-LRT counterpart PLHIV on treatment. The latter were either virally suppressed and had experienced an STI in the previous 12 months or were not virally suppressed for the full time period considered. Although some differences were observed in the second analysis (demonstrating the impact on our results of PLHIV – untreated or treated – with a detectable VL), these two analyses provided the same results: PLHIV meeting the biomedical criterion of HIV risk transmission did not report more sexual risk behaviours than their non-LRT counterparts.

## Discussion

Our results highlighted that PLHIV at LRT did not report more sexual risk behaviours than PLHW with higher risk of transmission. Indeed, LRT PLHIV in serodiscordant stable couples were more likely to be sexually abstinent, and both LRT MSM and women reported fewer sexual partners than their non-LRT counterparts. Moreover, rates of condom use within serodiscordant stable couples and during the most recent sexual encounter with a serodiscordant casual partner were, overall, similar between LRT and non-LRT groups. In line with results reported from a nationally representative sample of US PLHIV engaged in care [18], a majority of participants did not engage in condomless sex, either in serodiscordant couples or with the most recent non-HIV-positive casual partner. Virally suppressed MSM were significantly less likely to engage in sexual risk behaviour than their non-virally suppressed counterparts. Due to their cross-sectional design, neither the American study nor ours was able to evaluate changes in sexual risk behaviour before and after ART initiation, or VL suppression. However, both demonstrated that PLHIV with sUVL did not engage in sexual risk behaviour. The present results are also in line with those reported in several systematic reviews or meta-analyses on the association between ART and sexual behaviour. Although based on studies conducted in 1996 to 2003, before conclusive evidence that ART reduces HIV transmission risk, a 2004 meta-analysis comparing PLHIV on ART with a detectable VL to their counterparts with an UVL [12] highlighted that the rates of condomless sex were not higher in the latter group. The study showed that in general people (irrespective of serostatus) who believed that ART reduced HIV transmission were more likely to engage in condomless sex. Subsequent reviews in the developing world such as those by Kennedy *et al*. [13], Venkatesh *et al*. [14] and Kaye *et al*. [15] have reported either a transient increase or no increase in sexual risk behaviours following ART initiation. Furthermore, a recent review of studies conducted in developed countries provides additional support to the present study, as it underlined that PLHIV who were aware of their HIV-positive status and those on ART had decreased sexual risk behaviour [20].

It is well known that risk behaviour changes according to HIV disease stage [21]. Few studies have examined long-term risk behaviour changes in those living with HIV and on ART. Results from one such study, the Swiss HIV prospective cohort, showed an association between suppressive ART and increased unsafe sex among MSM and heterosexual women [17]. This result came two years after the publication of the Swiss statement when contrary results, similar to those of the present study, were published for the same Swiss cohort [22,23]. The present study's results are concordant with those from a sexual risk behaviour survey in 2007 among participants in a US prospective observational cohort ongoing since 1993, the HIV Outpatient Study, which reported no difference in condomless anal intercourse (despite it being very frequent) with partners of unknown or HIV-negative status among viremic or virologically suppressed MSM followed-up on in eight outpatient HIV clinics [24].

The 2013 French experts’ recommendations for PLHIV medical care advocated for ART initiation regardless of CD4 cell count, based on individual and public health benefits resulting from ART effectiveness [25]. In this context, understanding the relationship between viral suppression and sexual risk behaviour is particularly important. Results from a French cross-sectional community-based survey conducted in 2010 showed that 57% of participating PLHIV knew about the Swiss statement and 65% of the latter reported no change in condom use with HIV-negative partners. In that survey, awareness of the Swiss statement was significantly associated with having a UVL [26]. As the VESPA2 survey was conducted in 2011, it might be plausible that at least a portion of participants were aware of new biomedical HIV prevention approaches, however without this having any measurable impact on their sexual behaviour.

Our study has several limitations. First, the cross-sectional design of the VESPA2 survey did not allow us to evaluate sexual risk compensation over time, since this model concerns changes in risk perceptions and behaviour over time and requires knowledge of participants’ sexual behaviour before receiving ART or before achieving sustained UVL. Retrospective questions to investigate behaviour changes over time might have induced substantial recall bias, since participants in the study sample had been living with HIV for a rather long period of time. However such questions were not asked. Median duration time since diagnosis was 10.8 years and median time on ART was 7.6 years. Second, we lacked data about patients’ perception of transmission risk, awareness of the Swiss statement and perceptions of ART and of undetectable VL as prevention tools. This prevented any analysis about possible differences in transmission risk perception between having an undetectable versus detectable VL, or between having a recent STI versus no STI. Moreover, without this information we cannot exclude the possibility that participants on ART (i.e. 93% of those in participating in VESPA2) believed they were at low risk of transmission thanks to their treatment, but did not understand that their risk also depended on achieving sUVL and absence of STI. Third, sexual behaviours were assessed through face-to-face interviews, so underreporting socially unacceptable behaviours and inaccuracy of self-reported information cannot be completely excluded. Since both the Swiss study and our own assessed sexual behaviours through face-to-face interviews, discrepancy in results might be explained by the differences in both surveys’ designs (prospective cohort versus cross-sectional study) and in the national health care contexts where they took place (Switzerland versus France).

Despite its cross-sectional nature, our study is the first to be conducted among a nationally representative sample of PLHIV – including MSM, other men and women – that analyzes sexual risk behaviour according to the biomedical criterion of HIV transmission risk. The first wave of the VESPA survey was conducted in 2003. Despite a higher proportion of PLHIV with an UVL in 2011 with respect to 2003, the overall frequency of unsafe sex with serodiscordant steady partners, albeit showing some variation, remained comparable over time for MSM, other men and women [27–29]. This suggests that, at least in the French context, increased ART efficiency does not translate into increased sexual risk behaviour.

Risk compensation is currently a major issue regarding current and future users of new biomedical HIV prevention strategies [10,30,31]. The concern surrounding MSM is even greater mainly because HIV prevalence and incidence are still increasing in this population in most countries [32,33], including France, where MSM represented 43% of new HIV diagnoses in 2013 [11]. These two concerns underline the importance of including this topic in secondary HIV prevention interventions, in order to prevent any relapse into unsafe behaviours that might occur over time.

## Conclusions

Our results do not support the hypothesis of increased sexual risk behaviour among PLHIV presenting non-transmission biomedical characteristics in France. Indeed, some risk indicators suggested the opposite tendency. Positive long-term impacts of biomedical HIV prevention approaches, including ART, need to be accompanied by behavioural interventions, especially for high-risk individuals. Studies linking behavioural and clinical data are also necessary to assess the extent to which patients’ awareness of their VL might affect their sexual behaviour.

## Competing interests

None declared.

## Authors' contributions

MSM participated in the data analysis and interpretation of results and wrote the first draft of the manuscript. BD performed the statistical analyses. NL, MSM, FM and BS participated in the interpretation of results and contributed to the manuscript's review. FL, RDS, BS and MSM were also involved in the design and implementation of the VESPA2 survey. All authors approved the final version of the manuscript. All authors had full access to all of the data in the study and take responsibility for the integrity of the data, as well as the accuracy of the data analysis.
